# Allergic Comorbidity Is a Risk Factor for Not Attending Scheduled Outpatient Visits in Children with Asthma

**DOI:** 10.3390/children8121193

**Published:** 2021-12-16

**Authors:** Kalle Garpvall, Marie Hauerslev, Mads Marckmann, Mette N. Hermansen, Kirsten S. Hansen, Bo L. Chawes

**Affiliations:** 1 Department of Pediatric and Adolescent Medicine, Herlev and Gentofte Hospital, University of Copenhagen, Herlev, 2730 Copenhagen, Denmark; kgarpvall@gmail.com (K.G.); mariehauerslev@gmail.com (M.H.); mads.marckmann@gmail.com (M.M.); mette.northman.hermansen.02@regionh.dk (M.N.H.); kirsten.skamstrup.hansen@regionh.dk (K.S.H.); 2 Allergy Clinic, Herlev and Gentofte Hospital, University of Copenhagen, Gentofte, 2820 Copenhagen, Denmark; 3 COPSAC, Copenhagen Prospective Studies on Asthma in Childhood, Herlev and Gentofte Hospital, University of Copenhagen, Gentofte, 2820 Copenhagen, Denmark

**Keywords:** asthma, allergy, children, outpatient visits, adherence

## Abstract

Asthma is one of the most common chronic diseases in children globally. Previous studies have shown that not attending asthma primary care consultations is associated with poorer treatment adherence and increased risk of loss of asthma control on a short-term basis. Here, we investigated long-term patterns and predictors of not attending scheduled asthma outpatient visits during 5-years of follow-up in 146 children with asthma. Of the 146 children, 67 (46%) did not attend at least one scheduled appointment, amounting to a total of 122 (10.8%) missed of 1133 scheduled appointments. In a multivariate analysis adjusting for total scheduled visits in the 5-year period any allergic sensitization was a significant risk factor for not attending ≥1 scheduled appointment (aOR = 6.6 (95% CI, 1.3–39.7), *p* = 0.03), which was not the case for asthma treatment step or lung function. Furthermore, atopic predisposition decreased the risk of non-attendance (aOR = 0.36 (0.13–0.92), *p* = 0.04). We found no association between non-attendance, treatment adherence or loss of asthma control. This study highlights that allergic comorbidity, but not degree of asthma severity, identifies a group of children with asthma who are prone to not attend scheduled outpatient appointments.

## 1. Introduction

Asthma is a very common childhood disease and a rising incidence has been observed worldwide [1]. Previous studies have observed an association between not attending primary care consultations and subsequent risk of loss of asthma control and acute exacerbations [2,3]. Loss of asthma control can lead to admissions to hospital and deaths related to asthma [4] as well as a decrease in quality of life in patients and relatives [5], which makes it important to study patterns and predictors of not attending scheduled consultations for asthma in children. 

In a US cohort study of 5656 children with asthma, age 5–17 years, it was observed that patients who were younger, had higher use of asthma medication and lower BMI were more likely to attend their outpatient visits during a 12-month follow-up period [3]. Another large retrospective study of 1427 children 2–17 years of age showed that children attending primary care consultations with asthma within the past year were less likely to visit the emergency department (ED) with exacerbations [6]. Both these previous studies only had a follow-up period of one year, which is a short timeframe, particularly for children having mild-moderate disease burden, who are typically scheduled for 1–2 annual asthma consultations. 

In this study, we investigated long-term patterns and predictors of not attending scheduled asthma outpatient visits among 146 children and adolescents with mild to moderate asthma, who were followed during a 5-year period with the aim to identify risk factors of non-attendance. Subsequently, we analyzed the association between non-attendance, treatment adherence and loss of asthma control.

## 2. Materials and Methods

### 2.1. Study Design and Population

This is a retrospective investigation of a cohort of children with asthma who were recruited and followed since 2015 in our asthma outpatient clinic as previously detailed [7,8]. The inclusion criteria for the study were: (1) an asthma diagnosis established by a pediatrician in the clinic, and (2) one or more scheduled outpatient visits during 5-year of follow-up. We used the electronic medical records to obtain patient data, including baseline characteristics, clinical data, treatment adherence and loss of asthma control. A written consent form was signed by the parents/guardians prior to any data collection. 

Approval of the study was done by the Knowledge Center of data reviews in the Capital Region of Denmark on 8 July 2019, approval/case No. 19000557/5751312. 

### 2.2. Assessment of Non-Attendance of Scheduled Outpatient Visits

Data on the number of missed scheduled outpatient visits during the 5-years of follow-up were assessed with respect to the number of planned visits, i.e., missed + attended appointments, which were extracted from the electronic medical records. Some patients stopped attending the outpatient clinic either due to their asthma being well-controlled or turning 18 years of age. These patients had a shorter follow-up time but were not excluded from the analyses. 

### 2.3. Risk Factors of Non-Attendance 

The following potential risk factors at baseline for non-attendance were included in the study: sex (m/f); Caucasian ethnicity (yes/no); age (years); WHO z-score of body mass index (BMI); allergic rhinitis (yes/no); atopic eczema (yes/no); allergic sensitization (any, yes/no); house dust mites allergic sensitization (yes/no), cat/dog/horse allergic sensitization (yes/no), pollen allergic sensitization (birch/grass/mugwort, yes/no); food allergy (yes/no); asthma or eczema or allergy in first degree relative (yes/no); exercise challenge test (positive/negative, change in forced expiratory volume in first s (FEV1) > 10% from baseline, yes/no); physical activity beside physical education in school (yes/no), asthma controller treatment adherence (percentage of days covered (PDC) > 80%, yes/no); FEV1%-predicted; FEV1/forced vital capacity (FVC)-ratio (%); bronchodilator response (positive/negative, increase in FEV1 ≥ 12% from baseline); fractional exhaled nitric oxide (FeNO, ln(ppb)); GINA treatment step [8,9].

Spirometry was done while the child was sitting and wearing a nose clip with a MasterScope spirometer (Jaeger, Würzburg, Germany), repeated three times with a maximum of 5% difference between the best FEV1 values. The bronchodilator test was done measuring the % change in FEV1 from baseline 15 min after four puffs of salbutamol (Ventoline^®^, pMDI, 0.1 mg/dose (GlaxoSmithKline Pharma, Middlesex, UK). The exercise challenge test was done using a Valiant 2 Rehab XL motorized treadmill (Lode, Groningen, The Netherlands), slope 10%, speed 9 km/h for 6 min targeting a heart rate of ≥80% of the maximum expected for age, measuring the % change in FEV1 from baseline and 1, 3, 5, 10 and 15 min after exercise.

### 2.4. Loss of Asthma Control Measures

Loss of asthma control endpoints included: (1) being admitted to the pediatric ward or pediatric ED, (2) experiencing exacerbations not requiring admission, i.e., acute asthma symptoms treated with inhaled short-acting beta-2-agonist (SABA), (3) treatment with short course of oral corticosteroids (OCS) and (4) GINA treatment step-up prescribed by the outpatient clinic.

### 2.5. Statistical Analyses 

Baseline characteristics and risk factors for non-attendance were analyzed and compared between the groups of patients not attending at least one scheduled appointment during the follow-up period and patients attending all scheduled appointments. Analyses of baseline characteristics were done with Chi-square tests, Fisher’s exact tests and student’s *t*-tests. The risk factor analyses were modelled with logistic regression for binary data expressed as odds ratios (OR), linear regression for continuous data expressed as ß-coefficients and quasi-Poisson regression for count data calculating incidence risk ratios (IRR). All models are presented adjusted for the total number of scheduled appointments. R free software version 4.0.3 [9] and Microsoft Excel version 2013, Redmond, Washington, DC, USA [10] were used for the analyses and graphs.

## 3. Results

### 3.1. Baseline Characteristics

146 (94%) of the 155 children in the 2015 cohort had one or more scheduled outpatient visits during the 5-years of follow-up and were subsequently included in the analyses. The remaining nine children did not attend any scheduled visits despite getting called for a new appointment and being reminded by mail and were therefore excluded from the analyses. There were no significant differences when comparing the included and excluded children in baseline characteristics with the exception of a lower prevalence of any allergic sensitization and allergic rhinitis in the excluded children (Appendix A, Table A1).

The 146 included children had a mean age of 12.3 years (SD, 2.5), a total of 85 (58%) were males and 109 (75%) had Caucasian ethnicity. Among the included children, 104 (67%) had any allergic sensitization and 94 (64%) had allergic rhinitis. GINA treatment step at baseline was: median, IQR, 2 (1–3), and 47% were adherent to their controllers defined as a PDC > 80% (Table 1). 

Of the 146 included children, 67 (46%) did not attend at least one scheduled appointment, amounting to a total of 122 missed appointments (1.82 per patient). The 146 children had a total of 1133 scheduled visits in the 5-years of follow-up, i.e., 7.8 scheduled appointments per patient on average, corresponding to a 10.8% rate of missed appointments. The children attended a total of 1011 appointments, i.e., 6.9 on average per child. During the follow-up a total of 52 children turned 18 years and therefore stopped attending the outpatient clinic and further 10 children stopped attending the outpatient clinic due to their asthma being well controlled.

### 3.2. Risk Factors for Non-Attendance of Scheduled Appointments

Allergic comorbidity significantly increased the risk of non-attendance. Thus, in the univariate analyses we found that children not attending ≥1 scheduled outpatient appointment compared to children attending all scheduled appointments were more likely to have rhinitis (75% vs. 56%, OR 2.34, 95% CI 1.17–4.82, *p* = 0.02), any sensitization (85% vs. 59%, OR 3.88, 95% CI 1.78–9.07, *p* = 0.001), house dust mite sensitization (54% vs. 32%; OR 2.51, 95% CI 1.29–4.98, *p* = 0.007), pollen sensitization (69% vs. 48%, OR 2.36, 95% CI 1.21–4.72, *p* = 0.01) and showed a trend of more sensitization to furred animals (54% vs. 38%, OR 1.90, 95% CI 0.98–3.70, *p* = 0.06). These findings remained significant after adjustment for number of scheduled appointments during the 5-year follow-up period. 

In contrast, no significant differences were apparent for asthma severity (GINA treatment step), lung function (FEV1, FVC, FEV1/FVC ratio), bronchodilator response, bronchial responsiveness to exercise challenge test or FeNO level (Table 2).

Atopic predisposition in terms of 1st degree relatives with eczema showed a trend of decreased risk of non-attendance in both the unadjusted analysis and the analysis adjusted for No. of scheduled appointments (14% vs. 27%, aOR 0.39, 95% CI 0.14–0.99, *p* = 0.06, which was not the case for 1st degree relatives with asthma or allergy. No other risk factors of non-attendance of scheduled outpatient visits were identified in the univariate analyses (Table 2).

In a multivariate analysis including all univariate risk factors with *p* < 0.10, we found that children not attending ≥1 scheduled appointment compared to children attending all scheduled appointments had a higher risk of having any allergic sensitization (aOR 6.61, 95% CI 1.30–39.65, *p* = 0.03) and a lower risk of having eczema in a first degree relative (aOR 0.36, 95% CI 0.13–0.92, *p* = 0.04) (Table 3).

### 3.3. Non-Attendance, Treatment Adherence and Loss of Asthma Control

There was no difference in asthma controller treatment adherence among children not attending at least one scheduled appointment vs. children attending all appointments: PDC > 80%, 42% vs. 51%, aOR 0.61, 95% CI 0.25–1.43, *p* = 0.26, and no difference in treatment step-up according to GINA during the 5-years of follow-up: Number of steps, aß-coefficient 0.85, 95% CI 0.68–1.03, *p* = 0.14.

During the follow-up period, the 146 children experienced a total of 10 admissions to the pediatric ward, 56 exacerbations that required ED treatment or outpatient prescribed increase in SABA, and 13 short courses of OCS. There was no significant difference in either of the loss of asthma control endpoints among children not attending ≥1 scheduled appointment compared to children attending all scheduled appointments (Table 4). 

## 4. Discussion

In this study we found that among children with mild to moderate asthma, who are well-controlled and followed in our outpatient clinic, any allergic sensitization increased the risk of not attending scheduled visits, whereas atopic predisposition in terms of 1st degree relatives with eczema increased the likelihood of attendance. Additionally, we found that allergic rhinitis, sensitization to pollens and sensitization to house dust mites were also predictors of non-attendance in univariate analyses. In contrast, there were no significant findings for age, sex, BMI, ethnicity, GINA treatment step, FeNO level, lung function parameters or loss of asthma control.

A retrospective study of 5656 asthmatic US children examined visit attendance in the standard check-up program Well-Child Care (WCC) also found that comorbidity with atopic disease at any time increased the risk of partial attendance [3], which is supportive of this study’s findings. Lang et al. also identified other factors to be associated with increased risk of non-attendance including higher age, increased BMI and African American ethnicity, which is in contrast to our findings. However, our data indicated that increasing age and increasing BMI both increased the risk of non-attendance, but these findings were not significant. The influence of age was also studied by McGovern et al. in a population of 3895 US children with asthma aged 2–18 years, in which they found that younger children missed fewer primary care visits compared to older children [2]. Children of African American ethnicity were also found to have a higher risk of not attending WCC visits in a study by Wolf et al. of 152,418 children from low-income groups, but there was no attendance difference between patients, who were “White” and of “other races” [10]. The latter aligns with our finding of ethnicity not being a risk factor for non-attendance as we compared “Caucasian ethnicity” to “other ethnicity”. Interestingly, several studies have found that low-income groups are associated with low attendance rates to medical appointments [10,11]. Unfortunately, data on income level were not available for this study, which harvested data from the children’s electronic medical records, where income is not recorded. Another determinant, which could have been interesting to include in this study is health literacy, since it is known to be associated with poorer use of healthcare services [12]. 

There is a shortage of studies examining non-attendance to scheduled outpatient visits among the population of children with asthma, and thus a limited understanding of the reasons and risk factors driving not attending medical appointments for this group of patients with a very common chronic condition in childhood. However, a study of 127 children with inflammatory bowel disease found that adherence to blood samples was associated with a higher age of diagnosis, a shorter disease duration and exacerbations during a 3.2-year study period in the univariate analysis; however, in the multivariable analysis only shorter duration of disease was significant [13]. A US based study of 1771 children and adults up to age 30 years with type 1 diabetes, who had established care, also examined visit attendance over a two-year period, showing that those who missed ≥2 appointments had higher hbA1c, were of older age and were more likely to be female [14].

Our study identifies atopic comorbidity as the strongest predictor of non-attendance. We speculate that the phenotype of asthma that children with atopy suffer from may be more predictable for the child and guardians due to e.g., seasonal changes in symptoms during the pollen season, and thereby perceived as more easily manageable compared to non-atopic asthma with more unpredictable exacerbation due to e.g., infections. This enhanced understanding of the disease course in atopic asthma and the awareness that symptoms will improve as the seasons change, could lead to a feeling of less need for a scheduled outpatient visit. Another explanation could be that the children with atopic comorbidity are to a higher extent referred by their general practitioner to a tertiary asthma outpatient clinic at asthma debut, but in fact have less severe and less symptomatic asthma than the population without atopic disease, e.g., non-atopic severe wheeze [15,16]. 

We also found that eczema in a 1st degree relative led to a higher likelihood of attending all scheduled visits, which might be explained by the parents suffering from the same conditions and thus having a higher health literacy. It may be that parents who have atopic diseases themselves function as “enabling parents”, i.e., parents that have high attendance to scheduled visits, are more likely to fill their children’s prescriptions, reduce house dust mite and smoke exposure at home and other activities known to improve the child’s asthma control [17]. If indeed it improves asthma control as expected, it appears likely that children in these families will have less exacerbations and better overall quality of life [5]. 

Non-attendance has been shown associated with exacerbations of asthma and with ED visits in several studies. Smith et al. examined 1474 children aged 2–17 years with asthma and found that children attending primary care for asthma at least once in the previous 12 months had a lower risk of an ED visit than those who did not [6]. Additionally, the study found that filling at least one vs. none ICS prescription decreased the risk of an ED visit [6]. Another study of 5712 primarily African American children with asthma found that those with a lack of primary healthcare vs. those with good access to primary care had increased risk of asthma hospitalizations [18]. Further, a study of 3895 asthmatic children, age 2–18 years, showed that children attending vs. missing a primary care appointment had a decreased risk of a subsequent ED visit in the following 6 months [2]. 

A major strength of our study is the long follow-up time of 5 years since most studies of non-attendance only have up to 12 months follow-up. This is a short period of time considering that many children with mild to moderate asthma only see their pediatrician every 6 to 12 months. Additionally, our study population is rather homogenous with regards to severity of disease and socioeconomic status. Due to healthcare being free of charge in Denmark, some potential confounders with respect to inequality were eliminated in our study, where non-attendance is not caused by co-payments. 

A weakness of our study is the relatively small study population. This may explain the non-significant findings for e.g., age and BMI, and the lack of association between non-attendance, adherence to asthma maintenance therapy and risk of exacerbations. A contributing factor to this may be that the children in our study merely have mild to moderate asthma, are generally well-controlled and have fewer exacerbations compared to other studies [19,20,21]. ACQ and/or ACT would also have been helpful for assessment of asthma control, but such data was not collected. Finally, our study has a retrospective design, which leads to a risk of bias compared to prospective studies and randomized controlled trials.

## 5. Conclusions

This study shows that allergic comorbidity, but not degree of asthma severity or lung function identifies a group of seemingly well-controlled children with mild to moderate asthma, who are prone to not attend scheduled outpatient appointments during a 5-year follow-up period. This is an important reminder to clinicians following such children in order to implement strategies to improve attendance rates. 

## Figures and Tables

**Table 1:** Baseline characteristics stratified by children attending vs. not attending scheduled visits.

Baseline Characteristic	Missing Data	All Children (*n* = 146)	Children Attending All Scheduled Visits (*n* = 79)	Children not Attending ≥1 Scheduled Visits (*n* = 67)
Gender, male, *n* (%)	0	85 (58%)	44 (56%)	41 (61%)
Caucasian, *n* (%)	0	109 (75%)	59 (75%)	50 (75%)
Age, years, mean (SD)	0	12.3 (2.5)	12.1 (2.5)	12.5 (2.5)
BMI, WHO z-score, mean (SD)	1	0.06 (1.05)	0.02 (1.02)	0.1 (1.09)
Physically active, *n* (%)	17	110 (85%)	58 (85%)	52 (85%)
GINA treatment step, baseline, median (IQR)	4	2 (1–3)	2 (1–3)	2 (1–3)
Adherence, PDC > 80%, *n* (%)	56	42 (47%)	22 (51%)	20 (42%)
Rhinitis, *n* (%)	0	94 (64%)	44 (56%)	50 (75%)
Eczema, *n* (%)	4	51 (36%)	28 (37%)	23 (35%)
Sensitization, any, *n* (%)	0	104 (71%)	47 (59%)	57 (85%)
Sensitization, house dust mites, *n* (%)	0	61 (42%)	25 (32%)	36 (54%)
Sensitization, furred animals, *n* (%)	0	66 (45%)	30 (38%)	36 (54%)
Sensitization, pollen, *n* (%)	0	84 (56%)	38 (48%)	46 (69%)
Food allergy, *n* (%)	2	17 (12%)	11 (14%)	6 (9%)
Asthma predisposition, *n* (%)	19	58 (46%)	33 (49%)	25 (42%)
Allergy predisposition, *n* (%)	22	70 (56%)	35 (52%)	35 (61%)
Eczema predisposition, *n* (%)	23	26 (21%)	18 (27%)	8 (14%)
FEV1%-predicted, baseline, mean (SD)	0	88.6 (11.4)	89.2 (10.4)	87.8 (12,5)
FEV1/FVC-ratio, mean (SD)	0	82.1 (7.3)	82.4 (7.2)	81.7 (7.5)
FeNO, ppb, median (IQR)	6	15.5 (10–27)	14 (9–27)	18 (12–28)
Positive exercise challenge test, *n* (%)	0	42 (28%)	27 (34%)	15 (22%)

**Table 2:** Univariate risk factor analysis of not attending scheduled asthma outpatient appointments.

Risk Factor	Not Attending ≥1 vs. Attending All Scheduled Apointments					
	Unadjusted Analysis			Adjusted for No. of Scheduled Visits		
	OR	95% CI	*p*	aOR	95% CI	*p*
Sex (male vs. female)	0.80	0.41–1.54	0.50	0.82	0.42–1.61	0.57
Caucasian (yes vs. no)	1.00	0.47–2.12	0.99	1.11	0.51–2.38	0.79
Physically active (yes vs. no)	0.99	0.37–2.69	0.99	1.06	0.39–2.92	0.90
Rhinitis (yes vs. no)	2.34	1.17–4.82	0.02	2.21	1.09–4.60	0.03
Eczema (yes vs. no)	0.92	0.46–1.82	0.80	0.93	0.46–1.87	0.84
Sensitization, any (yes vs. no)	3.88	1.78–9.07	0.0005	3.65	1.66–8.61	0.002
Sensitization, house dust mites (yes/no)	2.51	1.29–4.98	0.007	2.38	1.21–4.77	0.01
Sensitization, furred animals (yes vs. no)	1.90	0.98–3.70	0.06	1.83	0.94–3.62	0.08
Sensitization, pollen (yes vs. no)	2.36	1.21–4.72	0.01	2.19	1.11–4.41	0.03
Food allergy (yes vs. no)	0.61	0.20–1.70	0.36	0.64	0.21–1.80	0.41
Asthma predisposition (yes vs. no)	0.80	39–1.62	0.54	0.81	0.40–1.67	0.58
Allergy predisposition (yes vs. no)	1.45	0.71–3.00	0.31	1.43	0.69–3.00	0.33
Eczema predisposition (yes vs. no)	0.44	0.16–1.07	0.08	0.39	0.14–0.99	0.06
Positive exercise challenge (yes vs. no)	0.56	0.25–1.20	0.14	0.54	0.24–1.19	0.13
Age (years)	1.07	0.94–1.22	0.34	1.07	0.94–1.23	0.31
BMI (WHO z-score)	1.10	0.81–1.50	0.55	1.10	0.80–1.50	0.56
GINA baseline step (1, 2, 3, 4–5)	1.12	0.80–1.57	0.51	1.12	0.80–1.59	0.52
FEV1 baseline (%-predicted)	0.34	0.02–6.11	0.46	0.34	0.02–6.46	0.47
FEV1/FVC baseline ratio	0.30	0.003–27.25	0.60	0.30	0.0029–29.35	0.94
Reversibility (%-change in FEV1)	0.99	0.95–1.02	0.44	0.99	0.95–1.02	0.45
FeNO baseline (ppb)	1.01	0.99–1.03	0.31	1.01	0.99–1.03	0.30
Log 2(FeNO baseline (ppb))	1.22	0.90–1.68	0.20	1.23	0.90–1.70	0.19

**Table 3:** Multivariate risk factor analysis of not attending scheduled asthma outpatient appointments.

Risk Factor	Not Attending Scheduled Outpatient Apointments					
	Unadjusted Analysis			Adjusted for No. of Scheduled Visits		
	OR	95% CI	*p*	aOR	95% CI	*p*
Rhinitis (yes vs. no)	0.52	0.12–1.84	0.33	0.51	0.12–1.82	0.32
Sensitization, any (yes vs. no)	6.60	1.30–39.42	0.03	6.61	1.30–39.65	0.03
Sensitization, house dust mites (yes vs. no)	1.11	0.42–2.96	0.83	1.12	0.42–2.99	0.82
Sensitization, furred animals (yes vs. no)	1.29	0,49–3.44	0.60	1.29	0.49–3.44	0.60
Sensitization, pollen (yes vs. no)	0.89	0.23–2.94	0.84	0.90	0.25–3.00	0.86
Eczema predisposition (yes vs. no)	0.35	0.13–0.92	0.04	0.36	0.13–0.92	0.04

**Table 4:** Association between non-attendance of scheduled appointments, treatment adherence and loss of asthma control.

Endpoint	Not Attending ≥1 vs. Attending All Scheduled Apointments					
	Unadjusted Analysis			Adjusted for No. of Scheduled Visits		
	OR	95% CI	*p*	aOR	95% CI	*p*
Adherence, PDC > 80% (yes vs. no)	0.70	0.30–1.60	0.40	0.61	0.25–1.43	0.26
	ß-coefficient	95% CI	*p*	aß-coefficient	95% CI	*p*
GINA treatment step-up (No. of steps)	0.92	0.75–1.11	0.46	0.85	0.68–1.03	0.14
	IRR	95% CI	*p*	aIRR	95% CI	*p*
Admissions (total No.)	1.04	0.51–1.60	0.89	0.92	0.45–1.45	0.78
Exacerbations (total No.)	0.97	0.63–1.32	0.86	0.86	0.55–1.19	0.43
OCS courses (total No.)	1.17	0.68–1.69	0.48	1.09	0.63–1.59	0.70

## Data Availability

All data are available on request from the corresponding author.

## Appendix A

**Table A1:** Comparison of included vs. excluded children.

Baseline Characteristic	Included (*n* = 146)	Excluded (*n* = 9)	*p*
Sex, *n* (%)			*p* = 1
Male	85 (0.58)	5 (0.56)	
Female	61 (0.42)	4 (0.44)	
Ethnicity, *n* (%)			*p* = 0.25
Caucasian	109 (0.75)	5 (0.55)	
Non-Caucasian	37 (0.25)	4 (0.44)	
Age, years, mean (SD)	12.25 (2.51)	12.70 (2.60)	*p* = 0.63
BMI, WHO z-score, mean (SD)	0.056 (1.05)	−0.63 (1.11)	*p* = 0.10
Comorbidity, *n* (%)			
Any allergic sensitization, no	42 (0.27)	7 (0.78)	*p* = 0.005
Any allergic sensitization, yes	104 (0.67)	2 (0.22)	
Allergic rhinitis, no	52 (0.36)	7 (0.78)	*p* = 0.027
Allergic rhinitis, yes	94 (0.64)	2 (0.22)	
House dust mite allergic sensitization, no	85 (0.58)	8 (0.89)	*p* = 0.086
House dust mite allergic sensitization, yes	61 (0.42)	1 (0.11)	
Cat/dog/horse allergic sensitization, no	80 (0.55)	8 (0.89)	*p* = 0.079
Cat/dog/horse allergic sensitization, yes	66 (0.45)	1 (0.11)	
Pollen allergic sensitization, no	62 (0.43)	7 (0.77)	*p* = 0.079
Pollen allergic sensitization, yes	84 (0.57)	2 (0.22)	
Eczema, no	91 (0.64)	8 (0.89)	*p* = 0.17
Eczema, yes	51 (0.36)	1 (0.11)	
Food allergy, no	127 (0.88)	9 (1.0)	*p* = 0.60
Food allergy, yes	17 (0.12)	0 (0)	
Lung function, mean (SD)			
FEV1% predicted	90.19 (11.22)	93.21 (11.18)	*p* = 0.45
FEV1/FVC-ratio	82.27 (0.96)	80.76 (1.09)	*p* = 0.67
FeNO, ppb	22.34 (20.77)	17.89 (18.59)	*p* = 0.51
GINA step at baseline, median (IQR)	2 (2)	2 (1.25)	*p* = 0.43
Exercise challenge test result, *n* (%)			*p* = 1
Negative	74 (0.63)	3 (0.60)	
Positive	43 (0.37)	2 (0.40)	
Regular physical activity, *n* (%)			*p* = 1
No	19 (0.15)	1 (0.14)	
Yes	110 (0.85)	6 (0.86)	
Asthma in 1st degree relative, *n* (%)			*p* = 0.73
No	70 (0.56)	6 (0.67)	
Yes	56 (0.44)	3 (0.33)	
Allergy in 1st degree relative, *n* (%)			*p* = 0.73
No	54 (0.44)	5 (0.56)	
Yes	70 (0.56)	4 (0.44)	
Eczema in 1st degree relative, *n* (%)			*p* = 1
No	96 (0.78)	7 (0.78)	
Yes	27 (0.22)	2 (0.22)	
Adherence, PDC > 80%, *n* (%)			*p* = 0.50
No	48 (0.53)	2 (1.0)	
Yes	42 (0.47)	0 (0)	
